# Accelerated loss of crystalline lens power initiating from emmetropia among young school children: a 2‐year longitudinal study

**DOI:** 10.1111/aos.15002

**Published:** 2021-08-19

**Authors:** Shuyu Xiong, Xiangui He, Padmaja Sankaridurg, Jianfeng Zhu, Jingjing Wang, Bo Zhang, Haidong Zou, Xun Xu

**Affiliations:** ^1^ Department of Preventative Ophthalmology Shanghai Eye Disease Prevention and Treatment Center Shanghai Eye Hospital Shanghai China; ^2^ Department of Ophthalmology Shanghai General Hospital Centers of Eye Shanghai Key Laboratory of Ocular Fundus Diseases Shanghai Jiao Tong University Shanghai China; ^3^ National Clinical Research Center for Eye Diseases Shanghai China; ^4^ Brien Holden Vision Institute Sydney Australia

**Keywords:** myopia, refractive development, crystalline lens, children

## Abstract

**Purpose:**

To determine the characteristics of crystalline lens with varying refractive errors and relationship with axial elongation in young school children.

**Methods:**

A total of 1465 children aged 6–8 years were examined annually for 2 years. Each participant underwent a series of ophthalmic examinations, including cycloplegic autorefraction, crystalline lens and axial length measurement. Crystalline lens power was determined, and factors associated with different refractive statuses were investigated.

**Results:**

Crystalline lens power decreased with time; reduction in lens power in Year 1 was greater in children with emmetropia (−0.69 ± 0.59 dioptre [D]) than in those with hyperopia (−0.49 ± 0.56 D) or myopia (−0.45 ± 0.55 D) (p < 0.001). Among the emmetropes, there were no differences in loss of crystalline lens power between those who remained emmetropic (−0.63 ± 0.59 D) and those who became myopic at Year 1 (−0.74 ± 0.61 D) and Year 2 (−0.77 ± 0.57 D, p > 0.05) visits. Among myopes at Year 1 visit, there was a greater reduction at Year 2 in those who had baseline emmetropia (−0.61 ± 0.51 D) than those who had baseline myopia (−0.26 ± 0.49 D, p < 0.001). The trend was similar among children of the same age. There was a significant correlation between changes in lens power and axial elongation in non‐myopia (β = −0.954, p < 0.001) and new myopia (β = −1.178, p < 0.001), but not in established myopia (β = −0.001, p = 0.539).

**Conclusions:**

There is accelerated loss of lens power in emmetropia and early stage of myopia. However, this loss is retarded when myopia persists and is accompanied by disappearance of the compensatory effect of lens power against axial elongation. These findings provide new insights into human refractive development.

## Introduction

The growth and changes in crystalline lens, especially in infancy and childhood, appear to be part of the coordinated growth and development process between the ocular biometric components (particularly the cornea, crystalline lens and eye length), leading to emmetropization (Iribarren, 2015). Among the optical elements, the crystalline lens appears to play a more dominant role than the cornea. Notably, the cornea has greater dioptric power and reaches a stable curvature and power by approximately 2 years of age; therefore, it is unlikely to contribute further. Thereafter, the change in crystalline lens power predominantly keeps pace with the change in axial length (AL) (Mutti et al., 2005; Guo et al., 2017). If the rate of axial elongation outpaces the changes in crystalline lens power, a myopic shift in mean refractive error occurs, eventually leading to the development of myopia (Mutti et al., 1998; Iribarren et al., 2012; Xiong et al., 2017).

Thus far, previous studies have not fully elucidated the nature of changes in crystalline lens power around the onset of myopia (Mutti et al., 2012; Rozema et al., 2019). The loss of lens power in established myopes appears to be less or reduced compared with that observed prior to the onset of myopia. However, whether this is a gradual process or induced based on threshold criteria remains uncertain and controversial (Iribarren et al., 2012; Xiong et al., 2017). Furthermore, because the change in crystalline lens power is related or linked to age, the effects of the interactions between age and refractive state on crystalline lens power warrant further investigation (Jones et al., 2005; Wong et al., 2010; Iribarren et al., 2012; Xiong et al., 2017). Additionally, the components and mechanisms involved in change in the crystalline lens power, around the onset of myopia remain speculative, although both refractive index and curvature are considered to play a role (Iribarren, 2015).

Therefore, we sought to better understand the role of crystalline lens power in refractive development, as well as the onset and progression of myopia. For this purpose, we investigated the interrelation of crystalline lens power with age, AL and refractive error state using ocular biometric data, namely AL, corneal power, anterior chamber depth (ACD), crystalline lens thickness and refractive error state. These data were gathered from a large sample of primary school children aged 6–8 years who were examined annually over a 2‐year period.

## Materials and methods

### Study participants

The study population included 1465 children enrolled in the Shanghai Time Outside to Reduce Myopia (STORM) trial. The STORM trial was a 2‐year, school‐based, prospective, cluster‐randomized study conducted from 2016 to 2018. Primarily, it was designed to determine the effect of outdoor time on the onset and progression of myopia. The methodology of this trial has been presented in detail elsewhere. In brief, school children (grades I–II) aged 6–8 years were enrolled and monitored annually over a 2‐year period (He et al., 2019). The trial was approved by the Shanghai General Hospital Ethics Committee and adhered to the tenets of the Declaration of Helsinki (ClinicalTrial. Gov Identifier: NCT02980445). The trial involved 6,295 children from 24 schools randomized to three groups (control and test groups I and II with outdoor intervention) from October 2016 to December 2018. Informed consent was provided by the parents or legal guardians of all children who participated in the trial.

### Data collection

All examinations were conducted at the school, and baseline biometric data were obtained from the school records. Each participant underwent an assessment at baseline and at annual intervals thereafter. The conducted procedures included the following: assessment of visual acuity with a retro‐illuminated Early Treatment of Diabetic Retinopathy Study chart (Guangzhou Xieyi Weishikang, Guangzhou, China; ambient room lighting at 4 m); slit‐lamp examination (66 Vision Tech, Suzhou, China); intraocular pressure check (non‐contact tonometer‐NT‐1000; Nidek, Tokyo, Japan); cycloplegic autorefraction (KR‐8900; Topcon, Tokyo, Japan); and AL measurements IOLMaster (version 5.02; Carl Zeiss, Jena, Germany). Measurements of the anterior and posterior radii of curvature of the cornea as well as ACD, lens thickness and central corneal thickness, assessed using a Pentacam (Oculus, Wetzlar, Germany), were performed in some of the children. The measurement was performed once for each child unless the quality of the measurement presented to be not ‘OK’, data of biometric measurements were then used in the following analysis only if the quality was ‘OK’. The examinations were conducted by experienced physicians who were trained in the aforementioned techniques prior to the initiation of the study.

Refractive error measurements and assessments of the crystalline lens curvature were conducted postcycloplegic examination. Briefly, cycloplegia was determined as follows: a single drop of 0.5% proparacaine hydrochloride (Alcaine; Alcon) was administered for topical anaesthesia, followed by two drops of 1% cyclopentolate (Cyclogyl; Alcon) administered 5 min apart. The pupil size and response to light was determined approximately 40 min after instillation. Cycloplegia was considered complete if the pupil size was large with no or negligible response to light.

### Data analysis

Of the original trial sample, 1465 children for whom crystalline lens thickness, corneal curvature, corneal thickness and ACD (using Pentacam) data were available for all visits (baseline and annual visits at Year 1 and Year 2) were included in the final analysis. Only data from the right eyes of the participants were analysed. The spherical equivalent (SE) was calculated as the sphere power plus half of the cylinder power in dioptres (D). Hyperopia, emmetropia and myopia were defined as SE ≥+1.00 D, between >−0.50 D and <+1.00 D, and ≤−0.50 D respectively (Guo et al., 2017; Rozema et al., 2019).

**Fig. 1:**
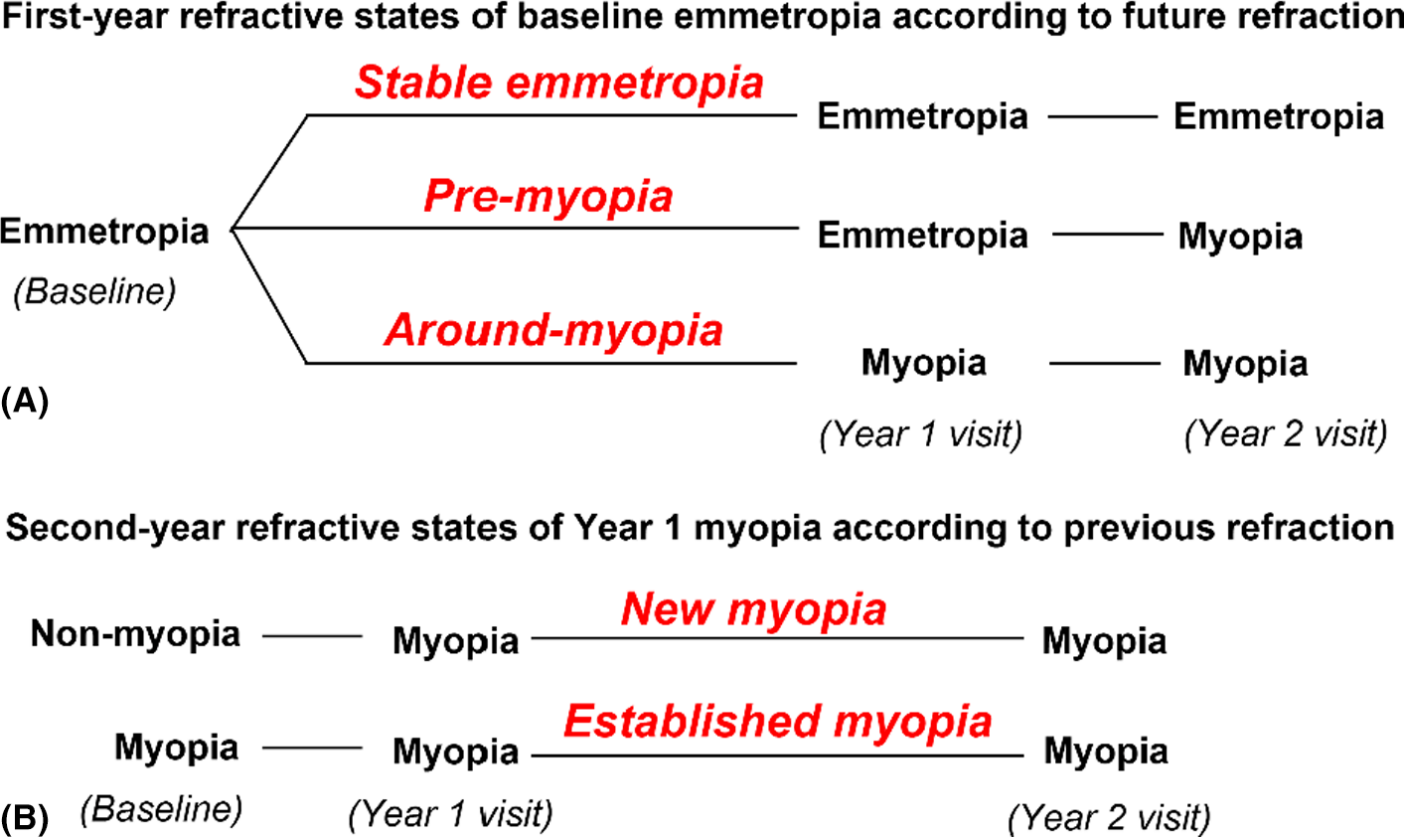
Refractive states for baseline emmetropia (A) and Year 1 myopia (B).

Baseline emmetropia was further categorized considering the future refractive state as (a) stable emmetropia (emmetropia at all visits, i.e. baseline, Year 1, and Year 2); (b) premyopia (emmetropia at baseline and Year 1, but myopia at Year 2 visit); and (c) around myopia (emmetropia at baseline, but myopia at Year 1 and Year 2 visits). Participants with myopia at Year 1 visit were categorized into the following states: (a) new myopia (non‐myopia at baseline, but myopia at Year 1) and (b) established myopia (myopia at both baseline and Year 1 visits). A detailed scheme is presented in Fig. 1.

The corneal power was calculated using the anterior and posterior corneal radii of curvature and a refractive index of 1.376, as proposed by Olsen and Manns et al., (IOVS, 2014; 55: ARVO E‐Abstract 3785) (Olsen, 1986).

Km,a = (nc−1)/ Rm,a

Km,p = (n−nc)/ Rm,p

K = Km,a + Km,p−Km,a * Km,p * CCT/nc.

where Km,a and Km,p are the mean anterior and posterior keratometric measurements respectively; Rm,a and Rm,p are the anterior and posterior corneal radii of curvature respectively; CCT is the central corneal thickness; and nc is the corneal refractive index.

The crystalline lens power was calculated using Bennett’s formula based on cycloplegic refraction, corneal power, ACD, lens thickness and AL (Bennett 1988; Rozema et al., 2011; Xiong et al., 2017), as shown below:

$$P_{L}=\frac{‐1000n(S_{\mathrm{CV}}+K)}{1000n‐\left(ACD+c_{1}T\right)\left(S_{\mathrm{CV}}+K\right)}+\frac{1000n}{‐c_{2}T+V}$$

where T is the lens thickness, V is the vitreous depth, *n* = 4/3 of the aqueous and vitreous indices, c1 = 0.596 and c2 = 0.358 as estimated using the Gullstrand–Emsley eye model. The SE refraction was defined as Scv = SE/(1–0.014 * SE). The effective ACD included the central corneal thickness, and the ACD as yielded by the Pentacam.

The parameters were presented as the mean ± standard deviation for the continuous variables. Intergroup differences were tested with Student’s *t*‐tests (between genders) or analysis of variance with post hoc tests (for refractive groups). Differences in biometric parameters (including lens power) between the baseline and each of the annual visits were compared using repeated‐measures analysis of variance by testing for sphericity. Changes in lens power and other biometric parameters (e.g. AL, SE, ACD, corneal power and lens thickness) between the first and second year of follow‐up were assessed using paired *t*‐tests. Correlations of the ocular biometric parameters and their changes were analysed using the Pearson correlation coefficients. Multiple regression analysis was performed to investigate the factors associated with changes in lens power among different refractive states.

## Results

### General characteristics

Table 1 presents the mean ocular biometric component data for the 1465 primary school children who participated in this study (mean age: 7.3 ± 0.6 years; *n* = 182 [6 years], 668 [7 years] and 615 [8 years]; 54.6% males). At baseline, older children had significantly reduced lens power, longer AL, more myopic SE, deeper ACD and a thinner crystalline lens (all p < 0.05), but no difference in corneal power (p = 0.317). Similarly, over the 2‐year follow‐up period, the crystalline lens power was reduced, the crystalline lens became thinner, the AL longer and the SE became less hyperopic/more myopic across all age groups. All changes were significant except for the change in ACD (p < 0.01) (Table 1). Additionally, although there were statistically significant changes in corneal power, these were practically negligible. All changes were greater in those that were younger at baseline.

**Table 1:** Ocular parameters at baseline and during a 2‐year follow‐up according to baseline age (Mean ± SD)

	Baseline	Year 1 follow‐up	Year 2 follow‐up	Changes		p‐value^‡^
				First‐year	Second‐year	
LP, D						
Total	26.66 ± 1.49	26.10 ± 1.46	25.61 ± 1.49	−0.56 ± 0.58*	−0.49 ± 0.52*	0.005
6 years (*n* = 182)	27.35 ± 1.46	26.65 ± 1.43	26.11 ± 1.46	−0.70 ± 0.59*	−0.54 ± 0.49*	0.007
7 years (*n* = 688)	26.77 ± 1.48	26.21 ± 1.43	25.70 ± 1.50	−0.56 ± 0.56*	−0.51 ± 0.54*	0.185
8 years (*n* = 615)	26.34 ± 1.42	25.83 ± 1.44	25.37 ± 1.44	−0.51 ± 0.59*	−0.46 ± 0.51*	0.102
p for trend^†^	<0.001	<0.001	<0.001	<0.001	0.062	
AL, mm						
Total	22.95 ± 0.73	23.21 ± 0.79	23.50 ± 0.87	0.26 ± 0.19*	0.29 ± 0.18*	<0.001
6 years	22.73 ± 0.64	23.06 ± 0.70	23.38 ± 0.78	0.33 ± 0.23*	0.33 ± 0.20*	0.923
7 years	22.88 ± 0.73	23.13 ± 0.78	23.41 ± 0.86	0.26 ± 0.19*	0.28 ± 0.18*	<0.001
8 years	23.09 ± 0.73	23.34 ± 0.80	23.64 ± 0.88	0.25 ± 0.18*	0.30 ± 0.17*	<0.001
p for trend^†^	<0.001	<0.001	<0.001	<0.001	0.061	
SE, D						
Total	+0.97 ± 0.99	+0.56 ± 1.22	+0.12 ± 1.48	−0.41 ± 0.47*	−0.45 ± 0.47*	0.005
6 years	+1.17 ± 0.88	+0.67 ± 1.13	+0.17 ± 1.39	−0.50 ± 0.55*	−0.50 ± 0.50*	0.930
7 years	+1.14 ± 0.94	+0.75 ± 1.15	+0.33 ± 1.42	−0.39 ± 0.47*	−0.42 ± 0.47*	0.286
8 years	+0.73 ± 1.03	+0.33 ± 1.29	−0.13 ± 1.55	−0.40 ± 0.45*	−0.47 ± 0.45*	0.001
p for trend^†^	<0.001	0.001	0.014	0.009	0.438	
ACD, mm						
Total	3.67 ± 0.21	3.72 ± 0.22	3.76 ± 0.22	0.04 ± 0.06*	0.04 ± 0.05*	0.407
6 years	3.60 ± 0.20	3.66 ± 0.21	3.70 ± 0.21	0.05 ± 0.05*	0.05 ± 0.06*	0.445
7 years	3.67 ± 0.21	3.71 ± 0.22	3.74 ± 0.22	0.03 ± 0.06*	0.04 ± 0.05*	0.488
8 years	3.72 ± 0.21	3.76 ± 0.22	3.80 ± 0.22	0.04 ± 0.06*	0.03 ± 0.05*	0.111
p for trend^†^	<0.001	<0.001	<0.001	0.020	0.006	
CP, D						
Total	41.28 ± 1.33	41.29 ± 1.33	41.25 ± 1.33	0.01 ± 0.24	−0.05 ± 0.22*	<0.001
6 years	41.21 ± 1.34	41.22 ± 1.34	41.15 ± 1.32	0.01 ± 0.24	−0.07 ± 0.22*	0.014
7 years	41.26 ± 1.34	41.26 ± 1.35	41.21 ± 1.34	0.00 ± 0.24	−0.04 ± 0.22*	0.009
8 years	41.32 ± 1.31	41.36 ± 1.32	41.32 ± 1.31	0.04 ± 0.25*	−0.04 ± 0.21*	<0.001
p for trend^†^	0.317	0.206	0.141	0.142	0.208	
LT, mm						
Total	3.48 ± 0.16	3.43 ± 0.16	3.40 ± 0.16	−0.05 ± 0.07*	−0.03 ± 0.06*	<0.001
6 years	3.54 ± 0.15	3.48 ± 0.15	3.45 ± 0.16	−0.06 ± 0.07*	−0.04 ± 0.07*	0.034
7 years	3.49 ± 0.15	3.44 ± 0.16	3.41 ± 0.16	−0.06 ± 0.07*	−0.03 ± 0.06*	<0.001
8 years	3.44 ± 0.16	3.40 ± 0.16	3.38 ± 0.16	−0.04 ± 0.07*	−0.02 ± 0.06*	<0.001
p for trend^†^	<0.001	<0.001	<0.001	0.052	0.001	

ACD = anterior chamber depth, AL = axial length, CP = corneal power, LP = lens power, LT = lens thickness, SD = standard deviation, SE = spherical equivalent.

* p < 0.01, compared between two visits using repeated‐measures analysis of variance with post hoc tests.

^†^ Comparison among age groups using variance analysis for trend.

^‡^ p‐value for comparison between the changes in the first and second year using a paired *t*‐test.

Crystalline lens power measurements at each of the three visits (i.e. baseline, Year 1 and Year 2) exhibited broadly Gaussian distributions, with a decrease in mean lens power over time (decrease in mean power of −0.56 ± 0.58 D at Year 1 versus baseline and −0.49 ± 0.52 D at Year 2 versus Year 1) (Table 1). Correspondingly, lens thickness decreased by −0.05 ± 0.07 mm and −0.03 ± 0.06 mm; AL increased by 0.26 ± 0.19 mm and 0.29 ± 0.18 mm; and refractive error demonstrated a myopic shift by −0.41 ± 0.47 D and −0.45 ± 0.47 D at Year 1 and Year 2 respectively.

### Baseline refractive state and changes in ocular components

**Fig. 2:**
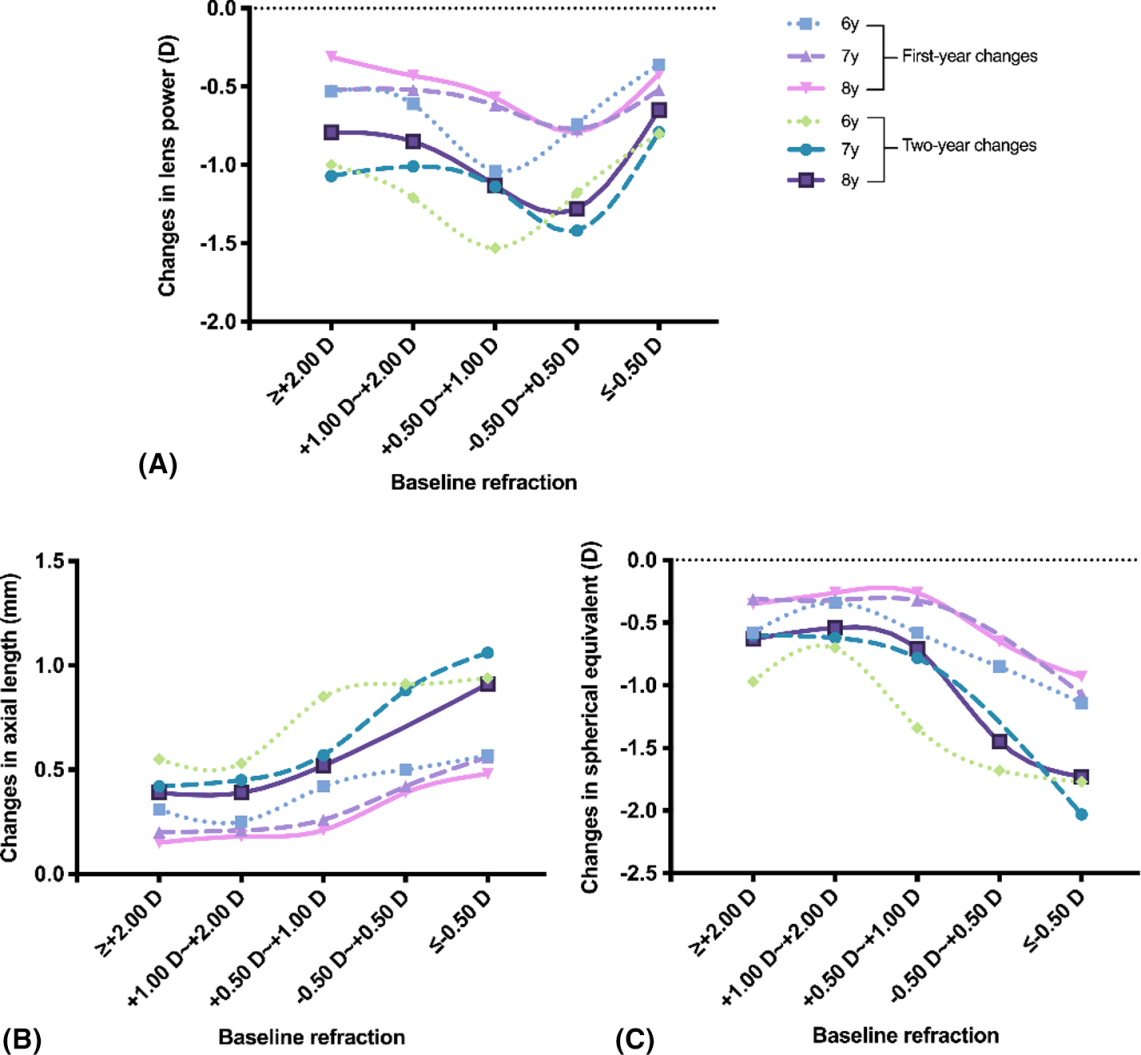
Changes in lens power (A), axial length (B) and spherical equivalent (C) in different age groups based on baseline refraction.

During the first year of the follow‐up period (Table 2), the loss in lens power was most pronounced in baseline emmetropes, followed by hyperopes and myopes (−0.69 ± 0.59 D, −0.49 ± 0.56 D and −0.45 ± 0.55 D respectively; p < 0.001), while changes in AL and SE were most pronounced in myopic eyes (p < 0.001) (Table 2). Participants with emmetropia with SE between −0.50 D and +0.50 D exhibited slightly greater reduction rate in lens power, but significantly faster axial elongation and greater myopic shift than those with SE between +0.50 D and +1.00 D (Table 2). The changing patterns of these parameters with baseline refraction were similar between different age groups, as illustrated in Fig. 2. Clinically, the differences in changes in ACD, corneal power and lens thickness were limited, despite reaching statistical significance for ACD (p < 0.001) (for corneal power: p = 0.472; for lens thickness: p = 0.049) (Table 2).

**Table 2:** Changes in ocular parameters based on different refractive status (Mean ± SD)

Refraction	LP, D		AL, mm		SE, D		ACD, mm		CP, D		LT, mm	
Baseline Refraction	First‐year	Second‐year	First‐year	Second‐year	First‐year	Second‐year	First‐year	Second‐year	First‐year	Second‐year	First‐year	Second‐year
Hyperopia												
Total (*n* = 854)	−0.49 ± 0.56	−0.49 ± 0.51	0.20 ± 0.13	0.23 ± 0.14	−0.31 ± 0.36	−0.31 ± 0.37	0.03 ± 0.06	0.03 ± 0.06	0.01 ± 0.24	−0.05 ± 0.21	−0.05 ± 0.07	−0.03 ± 0.06
≥+2.00 D (*n* = 149)	−0.47 ± 0.62	−0.52 ± 0.53	0.21 ± 0.14	0.23 ± 0.10	−0.37 ± 0.41	−0.31 ± 0.34	0.02 ± 0.06	0.03 ± 0.06	0.01 ± 0.27	−0.03 ± 0.22	−0.04 ± 0.07	−0.03 ± 0.06
+1.00 D to +2.00 D (*n* = 705)	−0.50 ± 0.55	−0.48 ± 0.51	0.20 ± 0.13	0.24 ± 0.15	−0.30 ± 0.35	−0.30 ± 0.38	0.03 ± 0.06	0.03 ± 0.05	0.01 ± 0.24	−0.05 ± 0.21	−0.05 ± 0.07	−0.03 ± 0.06
Emmetropia												
Total (*n* = 507)	−0.69 ± 0.59	−0.54 ± 0.52	0.31 ± 0.21	0.36 ± 0.19	−0.45 ± 0.53	−0.61 ± 0.51	0.05 ± 0.06	0.04 ± 0.05	0.02 ± 0.24	−0.05 ± 0.22	−0.06 ± 0.07	−0.03 ± 0.06
+0.50 D to +1.00 D (*n* = 328)	−0.65 ± 0.61	−0.54 ± 0.54	0.25 ± 0.19	0.32 ± 0.18	−0.32 ± 0.46	−0.49 ± 0.47	0.05 ± 0.06	0.04 ± 0.05	0.02 ± 0.24	−0.05 ± 0.22	−0.05 ± 0.07	−0.03 ± 0.06
−0.50 D to +0.50 D (*n* = 179)	−0.78 ± 0.55	−0.55 ± 0.50	0.41 ± 0.21^†^	0.44 ± 0.19^†^	−0.69 ± 0.58^†^	−0.82 ± 0.51^†^	0.06 ± 0.06	0.04 ± 0.05	0.03 ± 0.24	−0.04 ± 0.21	−0.07 ± 0.07	−0.03 ± 0.07
Myopia (*n* = 104)	−0.45 ± 0.55	−0.26 ± 0.49	0.51 ± 0.22	0.45 ± 0.16	−0.99 ± 0.54	−0.85 ± 0.44	0.05 ± 0.05	0.04 ± 0.05	0.03 ± 0.29	−0.05 ± 0.22	−0.05 ± 0.07	−0.01 ± 0.06
p‐value*	<0.001	<0.001	<0.001	<0.001	<0.001	<0.001	<0.001	0.056	0.472	0.878	0.049	0.121
Refractive changes for baseline emmetropia												
Stable emmetropia (*n* = 258)	−0.63 ± 0.59	−0.51 ± 0.49	0.18 ± 0.10	0.24 ± 0.11	−0.12 ± 0.29	−0.27 ± 0.30	0.04 ± 0.05	0.04 ± 0.05	0.02 ± 0.24	−0.05 ± 0.21	−0.05 ± 0.07	−0.03 ± 0.06
Premyopia (*n* = 125)	−0.77 ± 0.57	−0.56 ± 0.59	0.35 ± 0.13^‡^	0.49 ± 0.14^‡^	−0.50 ± 0.30^‡^	−0.95 ± 0.36^‡^	0.06 ± 0.06^‡^	0.04 ± 0.05	0.02 ± 0.22	−0.06 ± 0.24	−0.06 ± 0.07	−0.04 ± 0.07
Around myopia (*n* = 124)	−0.74 ± 0.61	−0.60 ± 0.51	0.55 ± 0.22^§^ ^,^ ^||^	0.50 ± 0.19^§^	−1.12 ± 0.46^§^ ^,^ ^||^	−0.96 ± 0.51^§^	0.06 ± 0.06^§^	0.04 ± 0.05	0.03 ± 0.26	−0.04 ± 0.22	−0.07 ± 0.07	−0.03 ± 0.07

Stable emmetropia: emmetropia at baseline, Year 1 and Year 2 visits; Premyopia: emmetropia at baseline and Year 1 visit and myopia at Year 2 visit; Around myopia: emmetropia at baseline and myopia at Year 1 and Year 2 visits.

ACD = anterior chamber depth, AL = axial length, CP = corneal power, LP = lens power, LT = lens thickness, SD = standard deviation, SE = spherical equivalent.

* Statistical significance among baseline refractions (over all categories) was tested using variance analysis.

^†^ p < 0.01, compared between those with SE within +0.50 D to +1.00 D and those with SE within −0.50 D to +0.50 D using a post hoc test.

^‡^ p < 0.01, compared between stable emmetropia and premyopic using a post hoc test.

^§^ p < 0.01, compared between stable emmetropia and around myopic using a post hoc test.

^||^ p < 0.01, compared between premyopic and around myopic using a post hoc test.

### Changes over the first year of the follow‐up period in baseline emmetropes

Baseline emmetropia was further categorized into stable emmetropia, premyopia and around myopia based on the future refractive state. Only data from the first‐year follow‐up period were analysed for the three categorizations. This is because, in the present study, it was impossible to determine the future states of emmetropes at the Year 1 visit based on the second‐year follow‐up data.

Of the 507 children with baseline emmetropia, 258 remained emmetropic over 2 years, whereas 124 and 125 children became myopic at Year 1 and Year 2 visits respectively. Irrespective of their future state from baseline, there were no differences in the loss of lens power between the aforementioned groups during the first‐year follow‐up period (−0.63 ± 0.59 D, −0.77 ± 0.57 D and −0.74 ± 0.61 D respectively) (Table 2). However, in those who became myopic, there were greater axial elongation and myopic shift in SE (0.55 ± 0.22 mm and 0.35 ± 0.13 mm versus 0.18 ± 0.10 mm for AL; −1.12 ± 0.46 D and −0.50 ± 0.30 D versus −0.12 ± 0.29 D for SE respectively). There was also greater change in ACD observed in those who became myopic.

### Changes over the second year of follow‐up in Year 1 myopes

Those who were myopic at Year 1 visit were categorized as new myopia and established myopia, according to their previous refractive state. Only data from the second‐year follow‐up period were analysed, because it is impossible to judge the previous states of myopes at baseline in the present study if the first‐year follow‐up data were used.

Among the 234 myopes at Year 1, 103 had baseline myopia and 131 were new myopes (or developed myopia during the first year of the follow‐up period). During the second year of the follow‐up period, the loss of lens power was greater in new myopes (−0.61 ± 0.51 D) versus established myopes (−0.26 ± 0.49 D, p < 0.001) (Table 3). There were no such differences in AL and SE observed between new myopic and persistent myopic eyes (0.51 ± 0.19 mm versus 0.45 ± 0.16 mm for AL; −0.97 ± 0.51 D versus −0.85 ± 0.44 D for SE respectively).

**Table 3:** Changes in ocular parameters based on refractive changes for myopia at first‐year visit (Mean ± SD)

Refraction	LP, D	AL, mm	SE, D	ACD, mm	CP, D	LT, mm
First‐year changes						
New myopia (*n* = 131)	−0.76 ± 0.60	0.56 ± 0.22	−1.15 ± 0.48	0.06 ± 0.06	0.04 ± 0.26	−0.07 ± 0.07
Established myopia (*n* = 103)	−0.45 ± 0.56	0.52 ± 0.21	−1.00 ± 0.53	0.05 ± 0.05	0.03 ± 0.28	−0.06 ± 0.07
p‐value*	<0.001	0.605	0.126	0.320	1.000	0.195
Second‐year changes						
New myopia (*n* = 131)	−0.61 ± 0.51	0.51 ± 0.19	−0.97 ± 0.51	0.05 ± 0.05	−0.04 ± 0.21	−0.03 ± 0.07
Established myopia (*n* = 103)	−0.26 ± 0.49	0.45 ± 0.16	−0.85 ± 0.44	0.04 ± 0.05	−0.04 ± 0.22	−0.01 ± 0.06
p‐value*	<0.001	0.037	0.117	0.798	1.000	0.141

New myopia: non‐myopia at baseline and myopia at Year 1 and Year 2 visits; Established myopia: myopia at baseline and Year 1 and Year 2 visits.

ACD = anterior chamber depth, AL = axial length, CP = corneal power, LP = lens power, LT = lens thickness, SD = standard deviation, SE = spherical equivalent.

* Statistical significance among new myopia and established myopia was tested using a post hoc test.

### Factors associated with changes in lens power

**Fig. 3:**
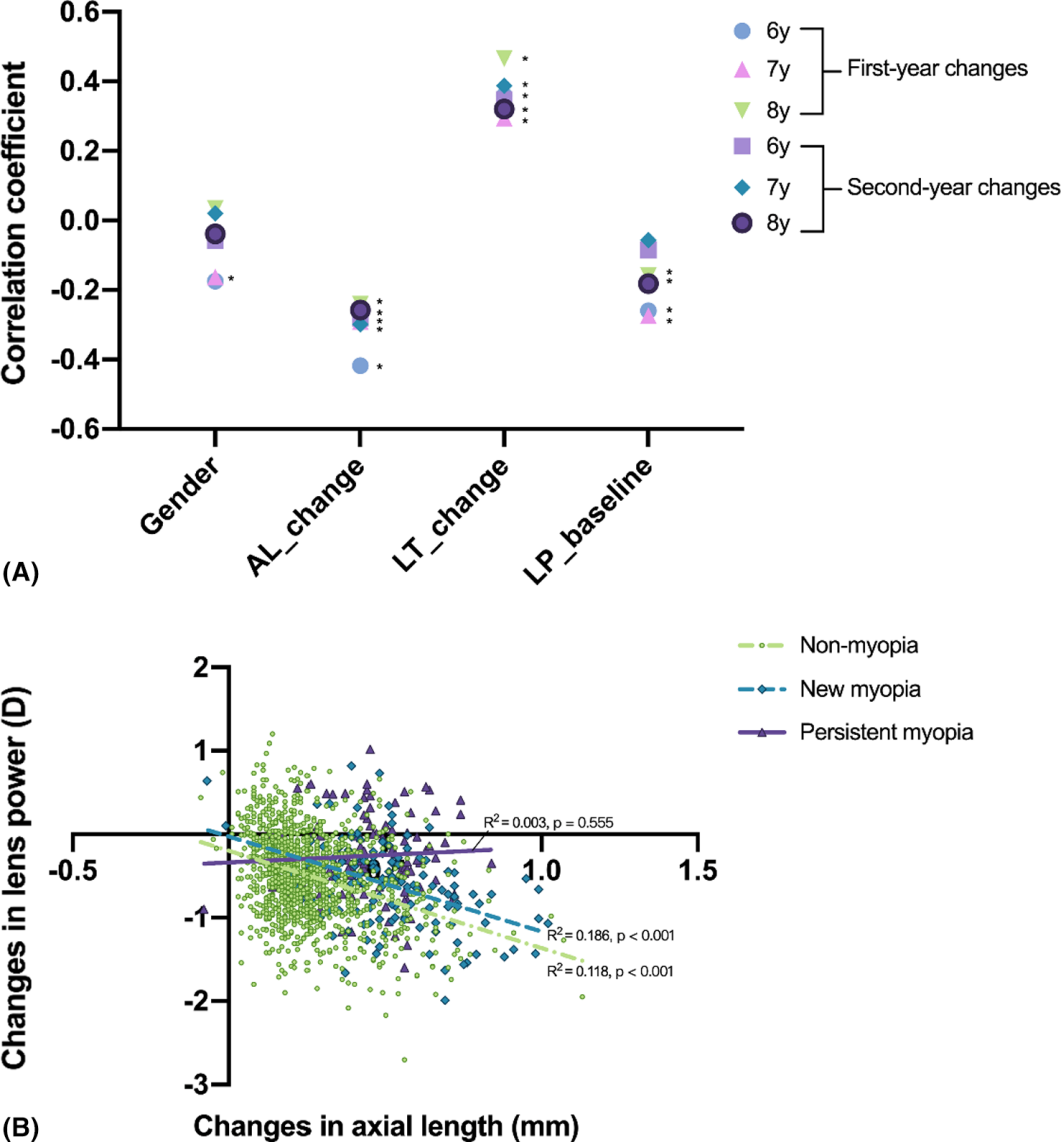
Factors associated with changes in lens power. (A) Correlation coefficient of both first‐ and second‐year changes in lens power with related factors for different age groups. ^*^Statistical significance of the variable. (B) Second‐year changes in lens power with axial length by different refractive states at Year 1 visit.

Factors associated with change in lens power during the second year of the follow‐up period were determined and found to be negatively correlated with Year 1 lens power, second‐year changes in AL, and positively correlated with second‐year changes in lens thickness (*r* = −0.120, −0.279, 0.359 respectively; p < 0.001). These relationships were similar for the first‐year changes and different age groups (Fig. 3A). The association between changes in lens power and changes in AL varied among refractive statuses. The mean change in lens power per millimetre increases in AL was −1.166 (−1.345 to −0.988) D, −1.139 (−1.554 to −0.723) D and 0.182 (−0.424 to 0.787) D for non‐myopes, new myopes and established myopes respectively (all p < 0.001, except for established myopes: p 0.553) (Fig. 3B).

In the multiple regression analysis, gender, changes in AL and lens thickness, baseline lens power and SE were independently associated with changes in lens power (Table 4). When stratified by refractive status at Year 1, there was a significantly negative correlation between the second‐year changes in lens power and changes in AL in non‐myopia (β = −0.954, p < 0.001) and new myopia (β = −1.178, p < 0.001), but not in established myopia (β = −0.001, p = 0.539). The changes in lens power were significantly correlated with changes in lens thickness and lens power at Year 1 in non‐myopia (lens thickness: β = 2.595, p < 0.001; lens power at Year 1: β = −0.059, p < 0.001) and established myopia (lens thickness: β = 2.551, p < 0.001; lens power at Year 1: β = −0.137, p = 0.001), but not in new myopia (lens thickness: β = 1.137, p = 0.068; lens power at Year 1: β = −0.011, p = 0.714). The adjusted factors are listed in Table 4.

**Table 4:** Multiple regression analysis for second‐year changes in lens power based on refraction at first‐year visit

Variables	Total (*n* = 1465)		Non‐myopia (*n* = 1231)		New myopia (*n* = 131)		Established myopia (*n* = 103)	
	Estimate (95% CI)	p‐value	Estimate (95% CI)	p‐value	Estimate (95% CI)	p‐value	Estimate (95% CI)	p‐value
Age_baseline	0.004 (−0.037 to 0.045)	0.834	0.000 (−0.044 to 0.043)	0.988	−0.105 (−0.235 to 0.025)	0.113	−0.018 (−0.202 to 0.166)	0.849
Gender	0.062 (0.012 to 0.113)	0.015	0.069 (0.015 to 0.122)	0.012	0.107 (−0.057 to 0.272)	0.199	0.105 (−0.085 to 0.295)	0.274
AL_change	−0.786 (−0.925 to −0.648)	<0.001	−1.030 (−1.203 to −0.858)	<0.001	−1.185 (−1.603 to −0.767)	<0.001	0.069 (−0.475 to 0.613)	0.802
LT_change	2.606 (2.228 to 2.984)	<0.001	2.595 (2.1854 to 3.005)	<0.001	1.137 (−0.086 to 2.361)	0.068	2.551 (1.186 to 3.915)	<0.001
LP_1st‐year	−0.063 (−0.080 to −0.045)	<0.001	−0.059 (−0.078 to −0.040)	<0.001	−0.011 (−0.068 to 0.047)	0.714	−0.137 (−0.216 to −0.058)	0.001

*R* ^2^ for total: 0.211; *R* ^2^ for non‐myopia: 0.242; *R* ^2^ for new myopia: 0.226; *R* ^2^ for established myopia: 0.238.

AL = axial length, CI = confidence interval, LP = lens power, LT = lens thickness.

## Discussion

The main finding of the present study was that the reduction in lens power varied during the refractive development among participants of the same age. The accelerated loss was initiated from emmetropia, more specifically SE <+1.00 D, regardless of the development of myopia in the future. Moreover, the loss of lens power continued after the onset of myopia, at least for the first year, and was gradually retarded when myopia persisted. In addition, there was no association between axial elongation and lens power loss in established myopia, whereas this association was significant in non‐myopia and new myopia.

The accelerated reduction in lens power initiating from emmetropia provided several important clues for the prevention of myopia. Firstly, hyperopia greater than +1.00 D is probably a safer state for young school children aged 6–8 years to preserve the compensating ability of the crystalline lens. According to our results, reaching emmetropia (SE <+1.00 D) earlier would result in earlier consumption of the lens power. This process was associated with a greater degree of uncompensated elongation during childhood, thus increasing the risk of myopia onset and progression. This is consistent with the common clinical intuition that early‐onset emmetropia is a major risk factor for the subsequent development of myopia (Zadnik et al., 1999; Flitcroft, 2014).

Secondly, emmetropia between +0.50 D and +1.00 D could be deemed as a warning refractive state, in which axial elongation could continue to be compensated by the reduction in lens power, maintaining the refraction relatively stable. Once emmetropia reaches −0.5 D to +0.50 D, AL increases further, and the reduction in lens power cannot fully compensate for the axial elongation, leading to the inevitable occurrence of myopia. Previous studies have also suggested an anti‐emmetropic effect of reduction in lens power. These findings proposed that mild hyperopia (approximately +0.50 D to +1.50 D), rather than the optically defined emmetropia (−0.50 D to +0.50 D), maybe the preferred end‐point for refractive development in humans (Sorsby et al., 1960; Morgan et al., 2010), while myopia could be the result of a failure in homeostasis (Flitcroft, 2013). Therefore, to prevent the onset of myopia, it is necessary to take measures, such as increasing outdoor time and decreasing time spent on digital devices, prior to the refraction reaching +0.50 D.

According to the results, this compensating process was similar among different age groups, with a slight decline in reduction rate observed with increasing age. This finding suggested that, apart from the natural development with ageing, the crystalline lens indeed changes causally as the refraction develops, but not coincidentally acting as a presentation of the relationship between age and refractive development, because older age is usually associated with more myopic refraction and higher prevalence of myopia in children (Ma et al., 2016). In addition, this process was also independent of the development of myopia in the future, with stable emmetropes exhibiting relatively less reduction in lens power than future myopes; this difference was mainly due to the less axial elongation in stable emmetropes (Xiang et al., 2012).

The present study further demonstrated that a rapid reduction in lens power continued after newly developed myopia, at least within the first year, which was retarded in those with persistent myopia. Moreover, Iribarren et al. suggested a significantly greater decrease in lens power for new myopia; however, the occurrence of such marked changes before or after the onset of myopia was not reported in that study (Iribarren et al., 2012). Our results implied that the crystalline lens continued to possess a compensatory ability at the early stage of myopia and underwent a more gradual reduction after the onset of myopia. This differed from the findings reported by Mutti et al., (2012), who did not indicate further loss of lens power immediately after the onset of myopia. The larger age range of participants (6–14 years) in that study may be a confounding factor. Previous studies had also observed that decreases in lens power were arrested or retarded after the age of 10 years (Mutti et al., 1998; Jones et al., 2005; Xiong et al., 2017). Therefore, it is difficult to determine whether the sudden cessation of lens power loss is due to increasing age or the onset of myopia.

There was a compensatory limit for the crystalline lens when myopia persisted. In such cases, the negative correlation between axial elongation and lens power loss was disappeared. These findings were consistent with those of previous studies (Cheng et al., 2021). Two mechanisms may explain this phenomenon. Firstly, the high consumption of lens power before and immediately after the onset of myopia could further result in significantly lower lens power in myopia versus non‐myopia (Iribarren et al., 2012; Xiong et al., 2017; Rozema et al., 2019). According to the results of the multiple regression analysis, lower lens power at baseline was related to less reduction in lens power. Secondly, this phenomenon may be associated with changes in anatomical features, including abnormally thicker posterior ciliary muscles and longer ciliary muscles in persistent myopia that restrict the equatorial growth of the eye, thereby causing further thinning of the crystalline lens (Bailey et al., 2008; Sheppard & Davies, 2010). The present findings also suggest limited thinning of the lens in persistent myopia.

Another interesting finding was that changes in lens thickness and baseline lens power were independently associated with changes in lens power in non‐myopia and persistent myopia, but not in new myopia. This suggests that different mechanisms underlying the compensating effect of lens power may be involved in the early stage of myopia development. Changes in the internal gradient index rather than the lens curvature may play a more active role in the compensating process immediately after the onset of myopia. Additionally, changes in lens power at the early stage of myopia may involve a more proactive process, unlimited by the baseline lens power.

Several limitations of this study should be acknowledged. Firstly, the aforementioned characteristics are applicable to children aged 6–8 years, prior to the stabilization of lens power at the age of 10 years. Further prospective studies are warranted to investigate the patterns of change around and after the onset of myopia for wider age range. Secondly, there may be some bias in the participant selection, because only those who agreed to undergo cycloplegic examination and had available data for lens thickness from all three visits were included, and children were taken from both control and intervention groups. However, the changing trends in lens power with refractive development and axial elongation, as well as other related factors, may not be affected. Finally, we propose some hypotheses on the mechanisms of lens power loss based solely on the current observations. Future animal experiments studying the internal structure of the crystalline lens or the morphology of the ciliary muscle would be helpful to uncover the exact compensatory and decompensated mechanisms of the lens.

In conclusion, the results of this study presented a clear pattern of change in lens power during refractive development. This pattern involved an accelerated loss of lens power initiating from emmetropia (regardless of the onset of myopia in the future and its natural development with ageing), continued with rapid loss in the early stage of myopia (if present), and was characterized by reduced loss when myopia persisted. Further research is warranted to elucidate the underlying mechanisms of this process. These findings may contribute to the current knowledge regarding human refractive development, which would facilitate the routine management of refractive development of young children and guide their normal growth to avoid the development of amblyopia and refractive error. The results may also contribute to the exploration of potential novel approaches for the prevention and control of myopia.
